# The Critical Role of PPAR*γ* in Human Malignant Melanoma

**DOI:** 10.1155/2008/503797

**Published:** 2008-05-11

**Authors:** Christian Freudlsperger, Udo Schumacher, Siegmar Reinert, Jürgen Hoffmann

**Affiliations:** ^1^Department of Oral and Maxillofacial Plastic Surgery, Tübingen University Hospital, Osianderstrasse 2-8, 72076 Tübingen, Germany; ^2^Department of Anatomy II: Experimental Morphology, University Medical Center Hamburg-Eppendorf, Martinistrasse 52, 20246 Hamburg, Germany

## Abstract

The past 30 years have only seen slight improvement in melanoma therapy. Despite a wide variety of therapeutic options, current survival for patients with metastatic disease is only 6–8 months. Part of the reason for this treatment failure is the broad chemoresistance of melanoma, which is due to an altered survival capacity and an inactivation of apoptotic pathways. Several targetable pathways, responsible for this survival/apoptosis resistance in melanoma, have been described and current research has focused on mechanism inactivating these pathways. As PPAR*γ* was shown to be constitutively active in several tumour entities and PPAR*γ* agonists extent strong anticancer effects, the role of PPAR*γ* as a possible target for specific anticancer strategy was investigated in numerous studies. However, only a few studies have focused on the effects of PPAR*γ* agonists in melanoma, showing conflicting results. The use of PPAR*γ* agonists in melanoma therapy has to be carefully weighted against considerable, undesirable side effects, as their mode of action is not fully understood and even pro-proliferative effects have been described. In the current review, we discuss the role of PPARs, in particular PPAR*γ* in melanoma and their potential role as a molecular target for melanoma therapy.

## 1. MALIGNANT MELANOMA AND MOLECULAR TARGETS IN MELANOMA THERAPY

**Figure 1:**
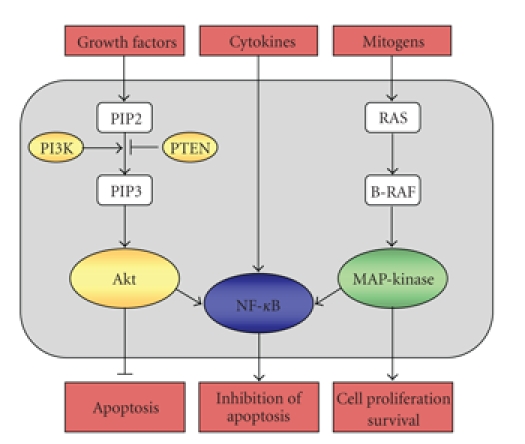
Activating pathways known to be constitutively active and contributing to the chemoresistance of melanoma cells. Over 60% of melanomas have activating mutations of B-RAF, 30% have lost PTEN expression, majority with NF-*κ*B activation.

Cutaneous malignant melanoma is the most aggressive form of skin cancer. Despite attempts to treat melanoma using a large variety of therapies, including immuno-, radio, and chemotherapies, survival remains very poor once the disease has spread to distant sites (median survival: 6–8 months) [1]. Systemic therapy, immunotherapy, or even biochemotherapy have failed to improve the survival of these patients. Until now, the only drug approved by the FDA for treatment of metastatic melanoma is the alkylating agent dacrabazine (DTIC), which results in clinical response only of 5–10% of cases when given as a single agent [2]. This treatment failure is mainly due to the notorious chemoresistance of melanoma cells. In contrast to other cancer cells, this chemoresistance of melanoma cells seems not to be acquired selectively following drug therapy, but to be more intrinsic in melanoma cells. Alteration of survival capacity and inactivation of apoptotic pathways are the molecular mechanisms responsible of conventional drug resistance in melanoma cells (see Figure 1). One example for a targetable pathway is the mitogen-activated protein kinase (MAP-kinase) pathway, which plays a crucial role in cell proliferation, invasion, and enhanced survival in diverse cancers [3]. A key player in the MAP-kinase pathway is B-RAF, a serin/threonine protein kinase acting as an oncogene [4]. The recent identification of activating mutations in B-RAF in over 60% cases of melanoma has offered the first opportunity for a rationale treatment program [3] and early clinical trials using the RAF kinase inhibitor BAY 43-9006 have been encouraging, being the first positive example of how targeted therapy can work in malignant melanoma. Other examples of targetable pathways in melanoma are the phosphoinositide-3-kinase (PI3K)/Akt pathway, which can be activated either through growth factors or loss of negative regulators of this pathway [5]. One of the most critical regulators of Akt (also known as protein kinase B) is the phophatase and tensin homologue (PTEN), which degrades the products of PI3K, preventing the activation of Akt [6]. In several studies it has been shown that up to 30% of melanoma, cell lines have lost PTEN expression [7]. Finally, Huang et al. investigated that the NF*κ*-B signaling pathway, acting as a key regulator of survival in cancer cells, is constitutively activated in melanoma cells [8]. In addition, a recent study has demonstrated that inhibiting NF*κ*-B activity, using the proteasome inhibitor bortezomib, reduced melanoma cell growth in vitro [9]. Although these targets seem to be attractive ones for melanoma therapy in the future, most of the findings in this area do not give a comprehensive picture which would warrant a review. As several studies have shown an antiproliferative effect of PPAR*γ* agonists on several tumour entities including melanoma, this review focuses on the role of the PPAR*γ* as a possible target in melanoma therapy.

## 2. PPAR*γ* AND PPAR*γ* AGONISTS

The peroxisome proliferator-activated receptors (PPARs) are ligand-activated transcription factors, belonging to the nuclear receptor superfamily [10]. Activated PPAR forms complexes with the retinoid receptor, which bind as a heterodimer to peroxisome proliferator response elements (PPREs) on the DNA, initiating transcription of downstream genes. The PPAR subfamily comprises three isoforms, PPAR*α*, PPAR*β*/*δ*, and PPAR*γ*, each showing a distinct distribution and ligand preference. While PPAR*α* is predominantly expressed in metabolically active tissue, like liver, kidney, skeletal muscle and brown fat [11], PPAR*δ* is expressed ubiquitously. PPAR*γ* is highly expressed in adipocytes, where it functions as a key regulator of adipocyte differentiation [12] and insulin-dependent glucose utilization [13]. Prostaglandin15-deoxy-Δ_12,14_-prostaglandin J_2_ (15d-PGJ_2_) is the most potent naturally occurring ligand for PPAR*γ* and the thiazolindinedione (TZD), also called glitazones, a class of antidiabetic, insulin-sensitizing drugs, are specific exogenous ligands for PPAR*γ*. The TZD family of PPAR*γ* agonists includes rosiglitazone, pioglitazone, ciglitazone, and troglitazone, rosiglitazone being the most potent agonist (Kd = 40 nM). In general, TZDs are selective for PPAR*γ* in concentrations of 10 *μ*M or less [14]. Recently, expression of PPAR*γ* has been demonstrated in tumor cells originating from various malignancies, including breast, colon, lung, gastric, pancreatic, prostate, and bladder cancer and its activation through PPAR*γ* agonists led to a significant decrease in proliferation of tumor cells in vitro [15–21], however, the exact mecahnisms underlying this effect are still being explored. As a consequence, PPAR*γ* has become a molecular target for potential anticancer drug development.

## 3. PPAR*γ* AND MELANOCYTES

Until now, there is little information on the PPAR subtypes and relative levels of PPAR protein expressed in human skin. The three PPAR subtypes have been investigated in human keratinocytes [22], and PPAR*γ* ligands have been shown to induce the expression of genes associated with keratinocyte differentiation in vitro [23]. In addition, Kang et al. showed the expression of all three PPAR subtypes in human melanocytes [24]. Immunocytochemistry showed that PPAR staining was mostly confined to the cytoplasm. Furthermore, proliferation of melanocytes was inhibited through administration of PPAR*α* (WY-14643) and PPAR*γ* (ciglitazone) agonists but not through PPAR*β*/*δ* (bezafibrate) agonists in a dose dependent manner at concentrations ranging between 0 and 100 *μ*M. The inhibitory effect of ciglitazone seemed to occur through induction of apoptosis, which was observed by the TUNEL method and flow cytometry [25]. Moreover, Lee et al. showed that pigmentation in melanocytes was accelerated with PPAR*α* and PPAR*γ* agonists, suggesting a possible role for PPAR*α* and PPAR*γ* in modulating melanocyte proliferation and differentiation (pigmentation) [26]. Eastham et al. investigated the expression of mRNA for PPAR*α*, PPAR*β*/*δ*, and PPAR*γ* in human melanocytes [27]. In addition, the natural PPAR*γ* agonist 15d-PGJ_2_ and the synthetic PPAR*γ* agonists ciglitazone and troglitazone inhibited the cell growth of human melanocytes, whereas the PPAR*α* agonists WY14643 and Leukotriene B_4_ had no effect on the proliferation of human melanocytes.

## 4. PPAR*γ* AND MELANOMA CELLS

Only a few studies have focussed on PPAR*γ* expression and effects of PPAR*γ* agonists in melanoma cell lines (summarized in Table 1). Mössner et al. investigated the expression of PPAR*γ* in four human melanoma cell lines MM-358, MM-201, MM-254 (established from lymph node metastasis of cutaneous malignant melanoma), and KAII (derived from a cutaneous nodular melanoma) [29]. In accordance with the immunocytochemistry of the melanocytes, staining was predominantly localized in the cytoplasm. In addition, the PPAR*γ* agonists 15d-PGJ_2_, troglitazone, and rosiglitazone dose-dependently inhibited the cell proliferation of all melanoma cells at concentrations between 0 and 50 *μ*M. As shown by flow cytometry, this antiproliferative effect was not mediated through induction of apoptosis, but rather by induction of G_1_ phase cell cycle arrest. Eastham et al. investigated the expressions of PPAR*α*, PPAR*β*/*δ*, and PPAR*γ* in human melanoma cells SK-mel28 and A375 [27]. Both melanoma cell lines express PPAR*α* protein levels 20–47% higher and PPAR*γ* protein levels 40–50% higher, respectively, than the normal human melanocytes. However, mRNA levels and protein levels for these receptors did not match. In addition, the natural PPAR*γ* agonist 15d-PGJ_2_ and the synthetic PPAR*γ* agonists ciglitazone and troglitazone inhibited the cell growth of the human melanoma cell line A375 in concentrations of 0–10 *μ*M, whereas the SK-mel28 cells were not affected in this concentration range. The PPAR*α* agonists WY14643 and leukotriene B4 had no effect on the cell proliferation of both cell lines. Placha et al. investigated PPAR*γ* expression in the melanoma cell lines WM35, derived from a primary tumour site, and A375, derived from a solid metastatic tumour. Furthermore, an antiproliferative effect of the PPAR*γ* agonist ciglitazone and 15d-PGJ_2_ in both melanoma cell lines was observed in concentrations of 10–15 *μ*M [30]. The antiproliferative effect of ciglitazone was mediated through induction of apoptosis, as evidenced by fluorescence microscopy. Núñez et al. showed an antiproliferative effect of 15d-PGJ_2_ on the melanoma cell line A375 at concentration of 16 *μ*M or higher, which was mediated through induction of apoptosis, while ciglitazone showed no growth inhibitory effect [31]. Our own results showed expression of PPAR*γ* in six different human melanoma cells MV3, Lox, MeWo, G361, FemX-1, and UISO-Mel6, which were established from primary malignant melanoma or metastatic melanoma lymph node [28]. Similar to the findings of Mössner et al., immunocytochemical staining of PPAR*γ* was mostly confined to the cytoplasm. The PPAR*γ* agonists rosiglitazone, pioglitazone, troglitazone, and ciglitazone all showed a dose-dependent antiproliferative effect on all melanoma cell lines tested at concentrations of 30 *μ*M or higher. This antiproliferative effect was due to a mechanism independent from apoptosis, which was shown by assessment of the nuclear morphology or by molecular analysis of DNA fragmentation. Interestingly, all four PPAR*γ* agonists showed an increase in cell proliferation of all six melanoma cell lines at concentrations of 3 *μ*M.

## 5. PPAR*γ*-DEPENDENT OR -INDEPENDENT EFFECTS OF PPAR*γ* AGONISTS IN MELANOMA CELLS

Several studies have documented various mechanisms for the antiproliferate effect of PPAR*γ* agonists, both being dependent or independent of PPAR*γ* activation, which holds also true for melanoma cells. Using a reporter gene assay, Eastham et al. showed that the PPAR*γ* agonists 15d-PGJ_2_ and ciglitazone stimulated PPRE reporter gene activity in a dose-dependent manner in B16 melanoma cells. This activity correlated with their ability to inhibit cell proliferation, hence a PPAR*γ*-dependent mechanism was postulated [27]. Simularely, Placha et al. investigated, that the apoptosis inducing effect of ciglitazone in human melanoma cells was clearly associated with the strong induction of transcription by endogenous PPAR*γ* through PPRE target sequences, as shown in the reporter gene assay system [30]. On the other hand, PPAR agonists have been reported to have nonreceptor mediated effects too. In our own studies, quantitative analyses of PPAR*γ* protein showed no correlation between the amount of the PPAR*γ* protein and the respective susceptibility of the melanoma cell lines towards PPAR*γ* agonists. Therefore, a PPAR*γ*-independent effect of PPAR-*γ* agonists was assumed [28]. In other cancer cells, resistance pathways which are constitutively activated in melanoma cells (Figure 1) were affected through PPAR*γ* agonist independent from PPAR*γ* activation. For example, 15d-PGJ_2_ has been shown to alter NF-*κ*B activity in hepatocellular carcinoma cells where 15d-PGJ_2_ induces apoptosis via caspase-dependent and -independent pathways [32]. In addition, Straus et al. showed a PPAR*γ*-independent repression of NF-*κ*B by 15d-PGJ_2_ through two mechanisms, inhibition of I*κ*B kinase (IKK) and inhibition of NF-*κ*B DNA binding [33]. The inhibition of NF-*κ*B by PPAR*γ* agonists through PPAR*γ*-independent mechanisms could be a possible way for the antiproliferative effect in melanoma cells, especially for the combination of the PPAR*γ* agonist rosiglitazone with bortzezomib, a potent inhibitor of NF-*κ*B, has led to an augmented antiproliferative effect on melanoma cells [34]. In addition, Han and Roman investigated that rosiglitazone inhibited the cell growth of human lung carcinoma cells through inactivation of PI3K/Akt pathway and increase of PTEN expression [35]. These changes were inhbited by GW9662, a potent antagonist of PPAR*γ*, suggesting that they depend upon PPAR*γ* activation. If this inactivation of the PI3K/Akt pathway by rosiglitazone also contributes to the antiproliferative effect of PPAR*γ* agonists in melanoma needs to be further elucidated.

## 6. CONCLUSION

The rapid increase in incidence of malignant melanoma has not been accompanied by better therapeutic options [36]. The past few years have seen great leaps in our understanding of the mechanism of drug resistance and cell survival in melanoma. Many reports have indicated the central role of PPAR*γ* in the control of malignant cell growth in various tumour entities including melanoma. In addition, evidence has been accumulated that PPAR*γ* is expressed in human melanoma cells and that PPAR*γ* specific agonists dose-dependently inhibited proliferation of melanoma cells [27–31]. However, these studies are inconsistent regarding the concentration of PPAR*γ* agonists initiating an antiproliferative effect and the mechanism underlying these growth inhibitory effects. In contrast to PPAR*γ*, PPAR*α* agonists were not shown to have antiproliferative effects on melanoma cells. Significant inhibition of melanoma cell proliferation did not occur until 20 *μ*M or higher concentrations of PPAR*γ* ligands were used. However, many natural and synthetic PPAR*γ* ligands loose their receptor selectivity at these concentrations [37]. Furthermore, conflicting evidence exists on the ability of PPAR*γ* agonists to promote tumour growth, depending on the cell model. For example, the PPAR-*γ* agonist troglitazone increased cell proliferation of breast cancer cells in low concentrations (<5 *μ*M), while higher concentrations of troglitazone (100 *μ*M) inhibited cell growth [38]. These investigations corroborate our published data in melanoma cells. The PPAR*γ* agonists rosiglitazone, troglitazone, pioglitazone, and ciglitazone showed an increase in cell proliferation of all six melanoma cell lines tested in low concentrations (3 *μ*M), however, in higher concentrations (>30 *μ*M) a significant growth-inhibitory effect was observerd [28]. In addition, Lucarelli et al. reported that troglitazone promoted the survival of osteosarcoma cells at concentrations of 5 *μ*M, through the activation of the PI3-kinase/Akt survival pathway (see Figure 1) [39]. Therefore, the administered dose of PPAR-*γ* ligands in clinical trials for melanoma therapy needs to be carefully defined and monitored closely.

In conclusion, the current studies concerning the role of PPAR*γ* in melanoma proliferation and progression report conflicting results. The concentrations inducing growth inhibitory effects in melanoma cells seem to be different depending on the PPAR*γ* agonist used and the melanoma cell employed. In particular, it remains to be further explored whether activation of PPAR*γ* itself or PPAR*γ*-independent effects of PPAR*γ* agonists contribute to the inhibition of melanoma cell growth. Although the antiproliferative effect of PPAR*γ* agonists in certain concentration ranges in melanoma is undisputable, more detailed information concerning the exact mechanisms seems to be necessary. However, PPAR*γ* might be a promising approach for target specific anticancer strategy in the treatment of melanoma.

## Figures and Tables

**Table 1:** Effects of PPAR*γ* agonists on melanoma cell growth.

Cell line	PPAR*γ* agonist	Concentration	Results	Mechanism of action	Reference
UISO-Mel6,	Rosiglitazone,	0.3–300 *μ*M	– Growth inhibition of all cell lines at	– Independent from apoptosis	Freudlsperger et al. [28]
MV3, MeWo,	pioglitazone,		30–300 *μ*M,		
G361,	ciglitazone,		– increase in cell proliferation at		
Lox	troglitazone		3 *μ*M		

MM-358,	15d-PGJ_2_,	0.1–50 *μ*M	– Growth inhibtion of all cell lines at 20–50 *μ*M	– Independent from apoptosis,	Mössner et al. [29]
MM-201,	troglitazone,			– induction of G_1_ phase	
MM-254, KAII	rosiglitazone			cell cycle arrest	

SK-mel28, A375	Ciglitazone,	0–10 *μ*M	– Growth inhbition only of A375 at 10 *μ*M	– Not investigated	Eastham et al. [27]
	troglitazone,				
	15d-PGJ_2_				

WM35, A375	Ciglitazone,	10–15 *μ*M	– Growth inhibition of all cell lines at	– Induction of apoptosis	Placha et al. [30]
	15d-PGJ_2_		10–15 *μ*M		

A375	Ciglitazone,	0–32 *μ*M	– Growth inhibtion of A375 at 16 *μ*M of 15d-PGJ_2_	– Induction of apoptosis	Núñez et al. [31]
	15d-PGJ_2_,		– no growth inhibition by ciglitazone		
